# Penile Anomalies in Adolescence

**DOI:** 10.1100/tsw.2011.38

**Published:** 2011-03-07

**Authors:** Dan Wood, Christopher Woodhouse

**Affiliations:** Adolescent Urology Department, University College London Hospitals, UK

**Keywords:** exstrophy, epispadias, micropenis, buried penis, hypospadias, phalloplasty, lymphoedema, penile fracture

## Abstract

This article considers the impact and outcomes of both treatment and underlying condition of penile anomalies in adolescent males. Major congenital anomalies (such as exstrophy/epispadias) are discussed, including the psychological outcomes, common problems (such as corporal asymmetry, chordee, and scarring) in this group, and surgical assessment for potential surgical candidates. The emergence of new surgical techniques continues to improve outcomes and potentially raises patient expectations. The importance of balanced discussion in conditions such as micropenis, including multidisciplinary support for patients, is important in order to achieve appropriate treatment decisions. Topical treatments may be of value, but in extreme cases, phalloplasty is a valuable option for patients to consider. In buried penis, the importance of careful assessment and, for the majority, a delay in surgery until puberty has completed is emphasised. In hypospadias patients, the variety of surgical procedures has complicated assessment of outcomes. It appears that true surgical success may be difficult to measure as many men who have had earlier operations are not reassessed in either puberty or adult life. There is also a brief discussion of acquired penile anomalies, including causation and treatment of lymphoedema, penile fracture/trauma, and priapism.

## INTRODUCTION – WHAT IS NORMAL?

It is difficult to fully understand how or exactly why men develop anxiety about penile size and function – the fact is that many do. There is a clear cultural and emotional background with many extreme practices described to enlarge or strengthen the male penis. For most, the anxieties are short lived and self-limiting. For some, resolution is achieved quickly with some medical reassurance, and for a few, an unswerving conviction results in so-called small penis syndrome[1,2]. There are, of course, some very important medical problems that require surgical treatment and in some cases, other treatments, including endocrine and psychological. For those that present in childhood, there are two real groups of patients: those who have had corrective surgery for conditions such as hypospadias, and those who have been told that either matters will all resolve once adolescence begins or that treatment can and will begin in adolescence.

There are many adolescent males who have genuine concerns and will present to an adolescent or a urology clinic, but the vast majority need time and explanation and no treatment. Occasionally, a teenage male may present with a rare diagnosis that is revealed by the presence of a micropenis[3]. Therefore, any such patient needs to be greeted with an open mind.

It important to understand that previous surgery may reinforce normal anxieties. This is exemplified in patients with hypospadias who may demonstrate a broad range of psychological outcomes, often unrelated to the surgical results. These concerns may be entirely mythical, such as the suggestion that 20% less men with hypospadias will marry compared to surgical controls. Others are based on good research, but are reports based on patients who had operations that gave an incomplete correction and are now outdated. It is also possible that regular review of the penis of young boys may induce an anxiety in those who are actually cured. It remains true that a good surgical repair may eliminate psychological impairment[2].

Other, rarer groups may have well-defined anomalies, such as bladder exstrophy or epispadias, and yet appear to develop strategies that put them in an adult group of successful high achievers[4].

In their review, Wylie and Eardley have given a comprehensive review of the papers referring to normal penile size[1]. The literature suggests that a stretched penile length of 12 cm is a sensible estimate of average. When the stretched penile length is <7 cm, the penis becomes definable as a micropenis.

## PENILE OUTCOMES IN BLADDER EXSTROPHY AND EPISPADIAS

One of the most anatomically challenging and dramatic congenital anomalies to confront a urologist is bladder exstrophy and epispadias. Their incidence is 1 in 30–50,000 and 1 in 100,000, respectively. There are approximately 20 live exstrophy births per year in the U.K. Over recent years, their care has been concentrated in specific centres to ensure both surgical and multidisciplinary expertise.

One objective for any male patient with exstrophy and/or epispadias should be to achieve a pendulous penis, with even corpora, resulting in a straight erection.

The discussion of outcomes has shifted dramatically in these patients. This is a significant testimony to the skills of those paediatric urologists who have reconstructed them in infancy. Historically, it was thought that the pubic diastasis was the sole reason for the shorter penis in exstrophy[5]. The classic appearance is that of a shorter penis with a wide girth. Based on an earlier model[6], the Johns Hopkins group have shown that there is also a significant reduction in corporeal tissue mass in the male exstrophy patient when compared to normal[7]. It is important to understand this basic piece of knowledge when counselling these men. For either surgeon or patient to expect a “normal” outcome is clearly unrealistic. A good result gives the patient with two evenly balanced corpora, a straight erection, a reasonable glans, and minimal recession of the mons. Particular problems seen in our own practice are:

- Asymmetric corpora – with one that is scarred and fibrotic and another that dominates. The fibrotic one acts as a bow-string on the other during erection, causing lateral curvature.
- Chordee – most commonly dorsal and considered to be an integral part of the epispadiac anomaly.
- Scarred and flattened glans.

Other authors have also identified the dorsal chordee and a small number of patients who have suffered sexual difficulty due to penile length[8], with some patients specifying that this is more limiting than deviation[9]. VanderBrink et al. present a large series relating to aesthetic outcome suggesting a 92% satisfaction rate in 65 patients. The difficulties with this series are that although follow-up time ranges from 1–29 years, we do not learn the ages at the time of primary surgery or current assessment, and there is no correlation with either sexual or psychological outcome[10].

In making an assessment of an epispadiac penis, it is important to consider current function in terms of sensation, erection, orgasm, and ejaculation. Any surgeon attempting reconstruction needs to understand that the position of the neurovascular bundle may vary considerably[11,12]. They may be far more lateral before operation and rotation of the corpora during previous reconstruction may further distort their position. Clearly, the unpredictability of their position renders them vulnerable during surgery. An anatomical assessment is usefully achieved with an artificial erection under general anaesthetic. Two large-bore butterfly cannulae are inserted (one into each corpora as there is no cross-circulation) and high-pressure saline is pushed in (300 mmHg) (see Fig. 1).

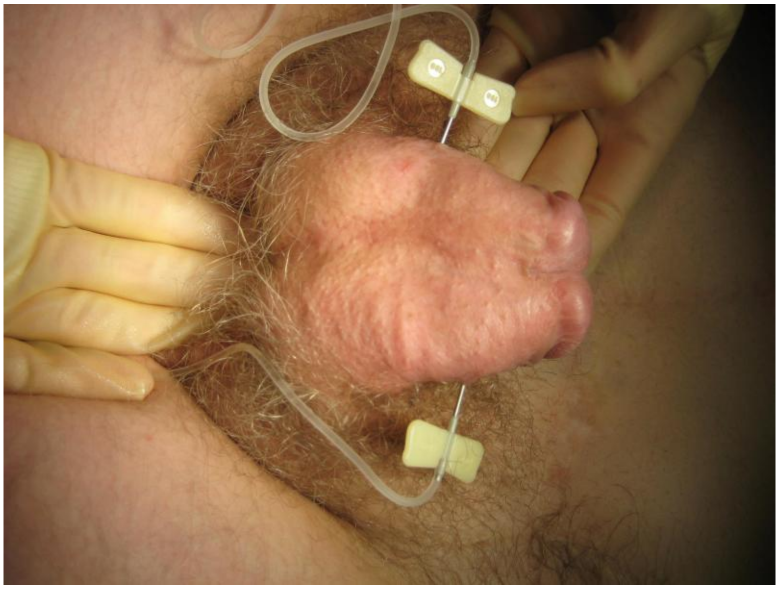

**FIGURE 1** Artificial erection being performed in a patient with bladder exstrophy.

However, it remains possible to disassemble the penis to correct chordee with a view to improving sexual function and cosmesis. This can be useful to improve a pre-existing chordee; realignment of uneven corpora may achieve this, in some cases, plication may be necessary. All patients need to be forewarned about penile shortening and loss of function, as would be standard practice with any corporal plication technique.

Other procedures, such as the penile Kelly operation, have been suggested as offering 1–2 cm of length improvement (Cuckow – personal communication). The corpora are completely detached from the inferior pubic rami and drawn anteriorly, maintaining a blood supply from the pudendal arteries. Although originally described nearly 30 years ago, it has been developed and applied well in highly specialised practice, such as that seen at Great Ormond Street, but has yet to establish its role in widespread practice[13]. Other authors have suggested that cosmetic outcomes may be less good due to a lack of pubic approximation with the Kelly procedure[14]. A note of caution is sounded about the risk of glanular and potential penile loss with the radical soft tissue mobilization that denotes this approach[15].

In their study to explore the psychological impact of bladder exstrophy on adolescents, Wilson et al.[4] found that specific anxieties relating to penile size and appearance were expressed. However, all participants in their study had begun to explore sexual behaviour through masturbation and all had a positive outlook in relation to sexual ambition, although there remained anxiety about negative reactions, bullying from peers, and finding a partner[4,16].

The penile function in these patients is enormously variable. For many older patients who have been diverted, the default position leaves the urethra as a seminal conduit that allows normal sensation with spontaneous erection, orgasm, and ejaculation.

## MICROPENIS

Micropenis provides a very different management challenge. Patients and parents present with two principle levels of anxiety. The first concerns the obvious, visible appearance of the penis and the second, a concern about diagnosis and treatment. Referrals may come from paediatricians, endocrinologists, and general practitioners; the source may or may not be able to give some indication about causation. Underlying diagnoses should be sought[3,17] to provide a fundamental management strategy. An isolated micropenis appears to occur more commonly with a gonadotrophin deficiency, while those associated with a lack of testosterone are often accompanied by hypospadias. Whilst micropenis may be idiopathic, recognised causes include:

- Primary testicular failure — e.g., anorchia, partial gonadal dysgenesis, Kleinfelter's syndrome
- Hypogonadotrophic hypogonadism — Kallman's syndrome, CHARGE syndrome
- Defects in testosterone action — partial androgen insensitivity, 5α reductase deficiency
- Developmental anomalies — aphallia, cloacal exstrophy

Another anxiety relates to long-term sexual function. The response of many patients and their parents is that they will simply not be able to pursue a normal sexual relationship and that an operation is the key to resolving this. The authors have spent many clinic consultations discussing these anxieties and trying to find a reasonable treatment path that is acceptable to both patient and treating surgeon. The data in Table 1 summarise seven series and suggest that the outcome for sexual function has been favourable. In this analysis, 95% of patients were having normal erections and 60% engaging in regular sexual intercourse. Thirty-eight percent of those included had psychological problems, which strongly suggests that careful counselling and psychological support are an essential component of management, and may allow some men to accept that they are able to achieve a meaningful and intimate relationship without surgical intervention[18,19,20,21,22,23,24].

**TABLE 1**
**Sexual Function with a Small Penis**

| | Reilly and Woodhouse | Miller and Grant | Money et al. | Husmann | Wisniewski et al. | Bin-Abbas et al. | Van Seters and Slob | Total |
|---|---|---|---|---|---|---|---|---|
| N | 20 | 19 | 9 | 20 | 13 | 8 | 3 | 92 |
| Gender identity disorder | 0 | | 2 | 0 | 0 | 0 | 0 | 2% |
| Erections | 20 | 15 | 9 | 20 | 13 | | 3 | 95% |
| Heterosexual | 20 | | 6 | 19 | 10 | | 3 | |
| Homosexual | 0 | | 3 | | 1 | | | |
| Bisexual | | | | 1 | 2 | | | |
| Regular sexual intercourse | 15 | 6 | | 12 | | 6 | 3 | 60% |
| Psychological problems | | 6 | 9 | 5 | 6 | 0 | | 38% |

For those patients who have not been assessed in childhood, it is sensible to arrange a karyotype and refer to an endocrinologist. Hormonal stimulation, especially with dihydrotestosterone, may produce some penile growth even after puberty. This can be given in a 2.5% gel formulation once per day; we would normally review after 6–8 weeks to assess the effect.

## PHALLOPLASTY

Techniques to create or augment a penis using either a graft or free-flap technique have been described for a number of indications. They have been applied in children and adolescents for a variety of disorders, including epispadias and micropenis[25]. Following an initial case study from Belgium[26], there are some long-term data about outcomes in the exstrophy group. Timsit and colleagues described six patients using a free-forearm graft technique, only one of whom had had penetrative sexual intercourse[27]. Following surgery, five described themselves as being satisfied with the outcome, with an increase in size from 4–6 cm stretched length to a mean of 12.6 cm. Cutaneous sensation was described as significant and three of the six were having intercourse following surgery.

Phalloplasty has also been used in cases of micropenis and aphallia.

In studies that have attempted to quantify the sensory outcome of phalloplasty, the radial artery forearm flap appears to be superior to the anterolateral thigh flap[28].

## BURIED PENIS

Young boys who are said to have a “buried penis” often have an entirely normal penis beneath excessive skin, fat, and connective tissue. In younger patients, there is a suggestion that inelasticity of the dartos fascia may result in the penis being tethered and not protruding as readily as it should. There are a few who have undergone circumcision and the resultant scarring leads to a similar effect and penile concealment as a result. Some authors have suggested that following newborn circumcision, this may improve from an initially buried appearance[29], suggesting that an initial conservative approach is justified.

In adolescent boys, the penis needs to be examined carefully. Gentle pressure around the penis will often push back the suprapubic tissues and reveal an underlying normal penis. This creates a dilemma for the surgeon about whether or not to operate and remove the suprapubic tissue. It is also possible to fix the base of the shaft to the pubic fascia and ventrally to accentuate the penoscrotal angle by fixing the subcutaneous tissue of the scrotum to the ventral surface of the penile shaft. Published series suggest that with a combination of adhesiolysis and lipectomy (suprapubic and lateral), erect length can be improved by up to 185%[30].

It seems sensible to apply some fairly rigid criteria before embarking on surgery in such patients. Firstly, other diagnoses, such as micropenis, need to be excluded. Secondly, patients need to understand that they have a normal penis, and they will be able to have normal intercourse and fertility without surgery. It is the authors’ opinion that they should definitely not have surgery until they have completed puberty, as with growth and development, the suprapubic fat pad may decrease considerably. It also seems sensible that they should receive appropriate advice and support about diet and exercise that may help them to resolve the appearance without an operation. This does not make surgery wrong, but these steps are important to avoid operating on patients either prematurely or unnecessarily. An augmentation phalloplasty may be what is offered with a z-plasty on the lateral and penoscrotal aspects of the shaft, with excision of the suprapubic fat and division of the penile suspensory ligaments. The Spyropoulos study relates to what is suggested to be micropenis, but Montorsi points out in the following editorial comment that the mean stretched length was 9.12 cm, which is above that meeting the definition of a micropenis[31]; therefore, the argument is raised about the need for surgery at all. This is an important point and needs to be considered very carefully in these patients. For those needing surgery, we would advocate delay until after puberty. Others would disagree and cite successful outcomes from their approach[32]. In paediatric practice, preputial flaps may act as useful grafts[33]. In circumstances of acquired buried penis, skin grafts and scrotoplasty may be useful[34].

## HYPOSPADIAS

There are two aspects to hypospadias in adolescent patients presenting for surgery: the present appearance and the prospects for improvement with surgery. Authors have suggested that hypospadias patients have an improvement in sexual and functional outcome that relates to their satisfaction with cosmesis[35]. A recent paper suggests that this may, in turn, relate to the original position of the meatus, i.e., the more severe the hypospadias, the more likely the patient is to find the cosmetic outcome unsatisfactory and, hence, for this to have a negative impact on future sexual function[36]. The assessment of sexual development has given rise to conflicting evidence. When comparing milestones to other surgical controls, there appeared to be no differences[37]. The other comparitors have been age of sexual debut, which tends to be later, and number of sexual partners, which tends to be fewer[35,38]. Whether these are good or bad markers of sexual outcome is open to debate. The timing of surgery also seems to be important. Evidence suggests that it will impact less on sexual function if performed before the age of 3 years[2].

Even now it is difficult to obtain a true measure of the outcome of surgical repair of hypospadias. Single-stage procedures are regularly cited as having a 90% success rate. The difficulty is, in part, that all patients should be assessed at least once during puberty and once when they have reached sexual maturity[39,40]. Virtually no men who are satisfied achieve this; hence, the true understanding of success or failure cannot be derived.

The other complicating feature of this disorder is the wide variation in surgical practice. There are over 200 named procedures for the repair of hypospadias, some of which are radically different and some of which are only minor deviations from each other. Many have been abandoned, but the patients on whom they were used are those that are available for follow-up in adult life.

If the potential for a lack of recognition of the true extent of the problem is added to this, it is easy to understand how and why failures happen; perhaps a little less easy to explain the most sensible way of avoiding them. What is universally accepted is the difficulty in managing those patients who fail in adolescence or adulthood[41,42,43,44]. In series of men presenting with a poor outcome, 36% were found to have one complication, with the majority (64%) having two or more complications, including residual hypospadias, chordee, stones, fistula, and diverticula. Success in repair of this very difficult problem ranges from 88 to 75%[41,42,43,44]. The need for a variety of techniques, both one- and two-stage, is also clear, although many revision and salvage procedures are staged.

## ACQUIRED PENILE ANOMALIES

The majority of this article deliberately discusses congenital anomalies and the effect these may have on adolescents. Whilst acquired anomalies are rare, they merit some brief discussion as they are often difficult, and some coherent thought processes can be helpful in assessment, explanation, and either treatment or explanation of likely treatment if a patient is being referred elsewhere.

### Lymphoedema

Penile lymphoedema (Fig. 2) is an uncomfortable and potentially disfiguring disorder. It may be congenital, in either an autosomal-dominant form (Milroy’s disease) or a sporadic form that occurs at puberty (Meigs’ disease)[45]. Whilst infective causes can be important, some authors suggest a trial of antibiotic therapy, with the use of steroids in acute or chronic exacerbations[46]. For many, the initial treatment is observation.

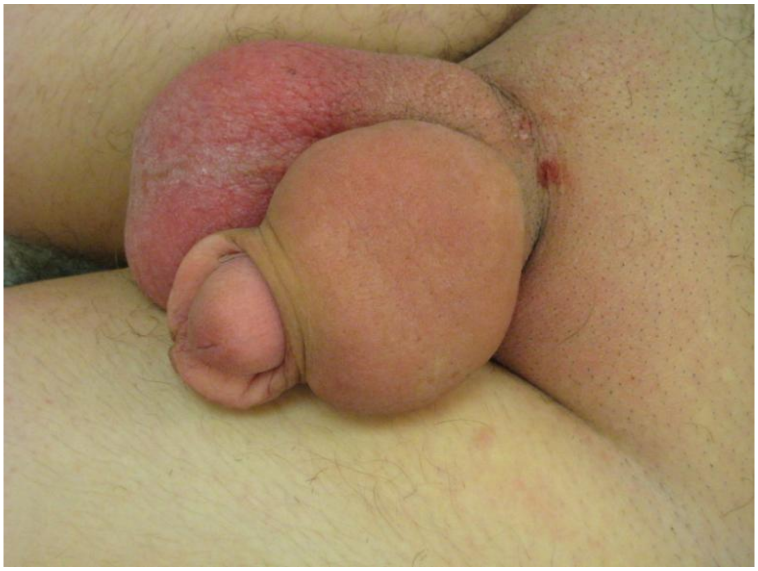

**FIGURE 2**. An example of penoscrotal lymphoedema.

A sensible classification of causes is as follows:

- Neoplastic
- Infectious
- Granulomatous
- Reactive
- Disorders of fluid balance, or
- Idiopathic[45]

Where there is no improvement with a conservative strategy, surgery becomes the only available treatment. This may involve incision of the skin and removal of all subcutaneous tissue down to deep fascia (Fig. 3); the overlying skin must be normal and not affected by sequelae of lymphoedema, such as abcesses or sinuses[45]. Recurrence may be a problem, either in the same or adjacent areas. Complete excision of the affected skin and replacement with split thickness skin grafts is also described. Some authors suggest this should only be used when other techniques have failed[47]. Others suggest that this gives better cosmetic results and should be the treatment of choice[45]. Neither technique is guaranteed to prevent recurrence and thus should be employed when underlying causes and other treatments have been excluded.

## Priapism

Stuttering or fulminant priapism is a distressing symptom sometimes associated with sickle cell disease, occurring in up to 50% of affected male patients[48]. If painful and low flow, permanent damage to erectile function may result. There is little evidence for a definitive treatment strategy. Clearly, basic principles of managing any sickle cell patient are important, including analgesia, oxygenation, and fluid resuscitation. Early involvement of the haematology team is also vital. Stilboestrol and alpha agonists have previously been tried[49], but a recent trial suggested that finasteride may be of some benefit in reducing episodes[50]. One publication suggests that sildenafil may be useful in the relief of a sickle cell priapism[51].

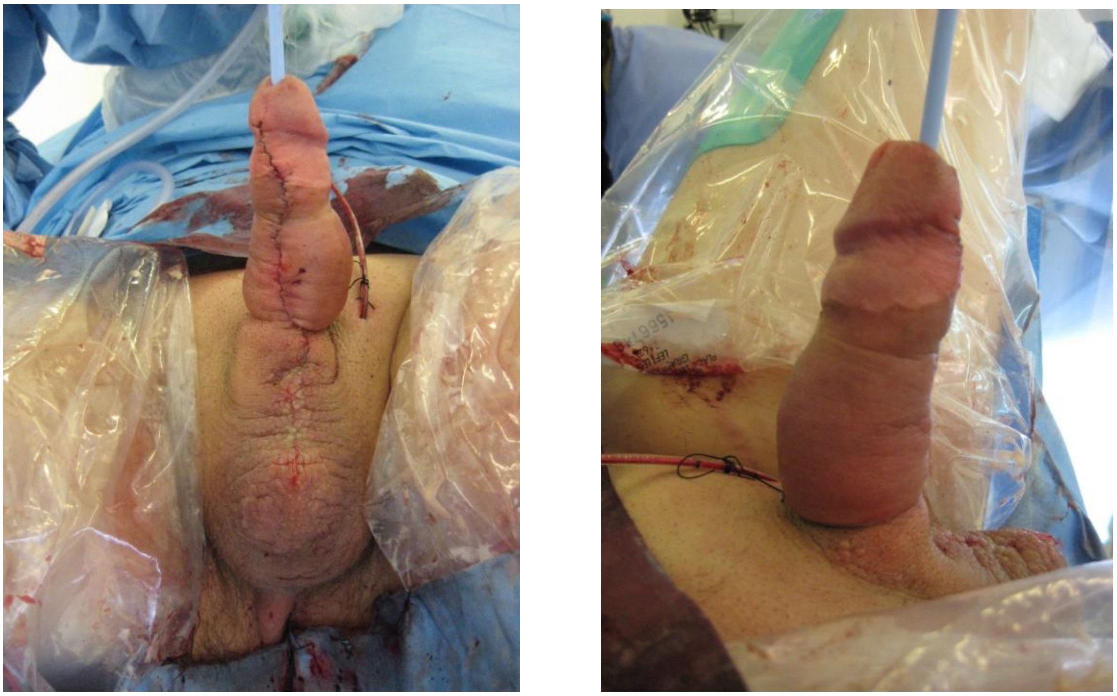

**FIGURE 3**. Immediate postoperative result of a subcutaneous excision for penoscrotal lymphoedema.

It is also worthy of note that in young men using cocaine, there is an increased incidence of priapism both when inhaled and applied topically to enhance sexual performance[52,53]. There also are case reports of PDE-5 inhibitors inducing priapism[54,55,56]. These incidences appear to relate to overdosing and probably do not affect the safety of the drug. It may be that combination with some other classes of drugs enhances this small risk[57].

## Penile Fractures and Trauma

Penile fracture classically presents following vigorous sexual intercourse during which a loud crack is heard followed by rapid detumesence. Other explanations include direct blunt trauma, sudden forced flexion, and rolling over in bed[58]! Classically, the penis swells and becomes discoloured by the haematoma, described as appearing like an aubergine. Corporeal injury is the most commonly recognised feature, however, urethral injury should be looked for as it may be found in up to 9%[58,59]. These two recently published series take in 292 patients and suggest presentation as early as 14 years (mean for both series 29 years); presentation ranging from 1 h to 45 days with good outcomes coming from prompt surgical repair using absorbable sutures. Penile trauma is also caused by other means, such as penetrating injuries (bullet or knife) and, rarely, self-inflicted injuries are encountered. The principles of management include assessment by an experienced penile surgeon, wound toilet, and debridement with viable tissue preservation and repair where possible[60].
